# Triamcinolone acetonide for aesthetic refinement in rhinoplasty for patients with thick skin: the FAN technique ‒ a pilot study

**DOI:** 10.1016/j.bjorl.2026.101786

**Published:** 2026-03-09

**Authors:** Ali Abdullah Alshehri

**Affiliations:** Surgery Department, College of Medicine, Najran University, Najran, Saudi Arabia

**Keywords:** Rhinoplasty, Skin, Thick, Triamcinolone, Patient satisfaction, Surgical outcome

## Abstract

**Objectives:**

Rhinoplasty outcomes can be affected by thick nasal skin, leading to prolonged swelling, delayed healing, and scarring. This study evaluates the effectiveness of Triamcinolone Acetonide (TA) administered via the Fine Administered Needle (FAN) technique to improve aesthetic outcomes in thick-skinned rhinoplasty patients.

**Methods:**

This pilot study included patients aged ≥18 with confirmed thick nasal skin who underwent rhinoplasty followed by TA injection using the FAN technique. A single subcutaneous injection at the nasal tip ensured optimal distribution. Swelling was assessed with a Visual Analog Scale (VAS), and patient-reported outcomes were evaluated using the Rhinoplasty Outcomes Evaluation (ROE) questionnaire.

**Results:**

The study included 17 patients with thick nasal skin (mean age 28.9-years; 58.8% female; 76.5% primary operations). ROE scores showed significant improvement, increasing from 16 (IQR4) to 22 (IQR2) by the third visit (p < 0.001). VAS scores for swelling decreased significantly in the infratip, tip-defining point, and supratip areas (p < 0.001). Subgroup analysis revealed no significant demographic differences. The procedure had a favorable safety profile with no major adverse effects or lasting complications.

**Conclusion:**

TA injection via the FAN technique is a promising adjunctive treatment for optimizing postoperative outcomes in thick-skinned rhinoplasty patients. This approach effectively reduces swelling, enhances nasal contouring, and improves patient satisfaction while maintaining an acceptable safety profile. Further studies with larger sample sizes and extended follow-up are warranted to validate these findings.

**Level of evidence:**

Level 4.

## Highlights

• ROE and VAS scores significantly improve post-injection.
• Single TA injection using FAN technique ensures even drug distribution.
• FAN technique provides safe, minimally invasive TA delivery.
• Tailored TA regimen offers sustained benefits in thick-skinned patients.

## Introduction

Rhinoplasty, or nose reshaping, is a common cosmetic surgery focusing on the nose’s external structure for both functional and aesthetic improvements.^1^ Patients may seek reductions in dorsal humps, narrowing of the midvault, or refinements of the nasal tip ‒ goals that are often achieved without compromising nasal airway patency or overall function.^2^ The aesthetic outcome is significantly influenced by skin thickness, which varies along the nose and is typically assessed through inspection and palpation of the nasal skin envelope.^3^^,^^4^ Thicker skin can obscure tip definition; when combined with weak cartilage, it may lead to tip ptosis or inadequate projection. In addition, thicker skin may mask the effects of other surgical maneuvers, increase postoperative edema, prolong healing time, and predispose patients to fibrosis or scarring.^5^, ^6^, ^7^

Postoperative challenges also include adapting thick skin to a restructured nasal framework, which may result in dead space that, over time, becomes filled with granulation tissue ‒ further contributing to edema and scar formation.^8^, ^9^, ^10^ To address supratip fullness after surgery, various strategies have been explored. One practical approach involves the injection of Triamcinolone Acetonide (TA), a corticosteroid that reduces fibroblast proliferation, inhibits collagen synthesis, and diminishes tissue fibrosis. TA further enhances collagen degradation by counteracting collagenase inhibitors and induces localized fat atrophy, with effects lasting up to six weeks at the injection site.^11^^,^^12^

Rather than employing multiple punctures, the Fine Administered Needle (FAN) technique uses a single needle prick ‒ inserted at the nasal tip and directed caudally within the subcutaneous plane ‒ to deliver the medication effectively while minimizing discomfort and complications. Despite its potential benefits, evidence supporting this approach remains limited. Accordingly, this pilot study aims to evaluate the effectiveness of TA injection via the FAN technique as an adjunct treatment for patients with thick nasal skin undergoing rhinoplasty by assessing its impact on postoperative swelling, aesthetic outcomes, patient satisfaction, and recovery using standardized assessment tools and patient-reported outcomes.

## Methods

### Study design and setting

This pilot study involved 17 patients with thick skin undergoing rhinoplasty at a single institution between May 2022 and October 2023. The study followed the ethical guidelines of the Helsinki Declaration (1964, revised 2013) and adhered to the STROBE Checklist.^13^^,^^14^ Ethical approval was obtained from the institution’s Research Ethics Committee. Informed consent was obtained from all participants, and data were anonymized.

### Eligibility criteria

Patients aged 18-years and older with thick skin and scheduled to undergo rhinoplasty were included in the current study. The diagnostic criteria for thick skin included the presence of oily skin, a positive pinch test indicating increased subcutaneous thickness, and large pores on the nasal tip skin, as described in previous literature.^15^^,^^16^ Additional factors such as dermal thickness and sebaceous gland hypertrophy were also considered in the assessment. All patients met the 'yes' criteria for each of the diagnostic indicators. Participants must have provided informed consent for their involvement in the research.

Exclusion criteria for the study included patients with active skin infections or systemic conditions that could impair healing and patients who had previously received corticosteroids, immunosuppressants, or vitamin A derivatives. Moreover, patients using isotretinoin prior to the study period were excluded.

### Study procedure

After obtaining informed consent, participants underwent a comprehensive preoperative evaluation adhering to clinical practice guidelines. This evaluation included a thorough medical history and physical examination.

Diagnostic imaging, primarily Computed Tomography (CT) scans as per institutional standard guidelines, was used to evaluate nasal anatomy and identify structural deformities and sinus pathology. Skin thickness was assessed clinically, without reliance solely on imaging. Additionally, laboratory tests and an anesthesia evaluation were conducted to ensure patient safety and facilitate optimal surgical planning. Each patient completed preoperative baseline assessments on the day of surgery.

All patients underwent open structural rhinoplasty procedures performed by a single experienced surgeon to minimize variability in technique, which is crucial for maintaining study validity.

The FAN technique was applied intraoperatively at the end of the rhinoplasty procedure, after suturing but before dressing placement. Although the nasal structures were dissected and opened during surgery, the Triamcinolone Acetonide (TA) injection targeted intact subcutaneous tissue at the nasal tip via the same puncture site used for surgical manipulation, ensuring precise delivery.

Intraoperatively, the entire dose of 40 mg (1 mL) of TA was infiltrated through a single puncture using an anticlockwise needle movement to ensure even distribution throughout the target area. No local anesthetic agent was administered prior to or during infiltration; instead, the area was irrigated with cold normal saline, which provided topical anesthesia by numbing the tissue surface. Only one injection puncture was made per patient, with the needle inserted once at the nasal tip, initially directed caudally toward the infratip area, then slightly withdrawn and redirected upward within the subcutaneous plane. Importantly, infiltration of TA was performed both during needle insertion and withdrawal, with the needle “dancing” beneath the skin to achieve uniform distribution of the corticosteroid across the subcutaneous tissues.

Postoperatively, a schedule for punctures was provided to all patients, along with full instructions regarding potential adverse effects and a structured plan that had been explained preoperatively.

**Fig. 1:**
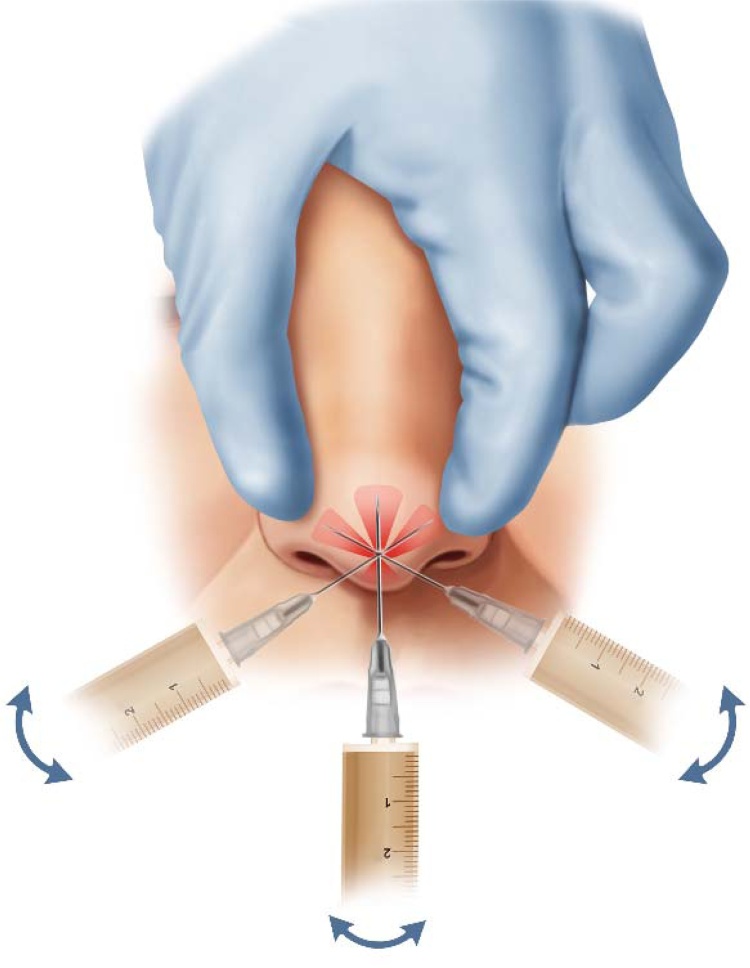
Administration of Triamcinolone Acetonide via the FAN Technique.

During postoperative follow-up visits, the FAN technique was employed in a structured manner (Fig. 1). Materials, including a 27 G fine needle, TA, and aseptic instruments, were prepared. The nasal area was cleaned, and the fine needle was inserted at the nasal tip, directed caudally, then slightly withdrawn and pointed upward in the subcutaneous plane. Monitoring for complications was conducted throughout the process. After the injection, gentle pressure was applied, and micro-pore tape was applied for up to 2 h per day for several days to control bleeding at the puncture site; this was not intended to reduce edema or improve healing. Post-procedure care instructions were provided, including monitoring for redness or swelling and avoiding strenuous activity. Follow-up appointments were scheduled to evaluate the injection’s effects and potential side effects.

### Assessment tools

Data collection included direct assessments and patient interviews to gather demographic information (age, sex, skin type) and details on triamcinolone formulation and dosage. Postoperative follow-up assessments measured swelling at specific anatomical points (tip defining point, tip projection, supra- and infratip areas) using the Visual Analogue Scale (VAS), recorded by the examiner for each anatomical site, and patient-reported outcomes were measured via the Rhinoplasty Outcome Evaluation (ROE) questionnaire. Assessments were conducted preoperatively and at three intervals during follow-up visits.

The VAS is a well-validated measure commonly used in clinical settings; it ranges from 0 (indicating no swelling) to 10 (indicating extreme swelling with skin discoloration).^17^^,^^18^ The ROE is another validated questionnaire available in multiple languages (including Arabic)^19^ that allows for a comprehensive assessment of rhinoplasty-related patient-reported outcomes and satisfaction. The ROE questionnaire consists of six questions with five answer options graded from zero to four, resulting in scores ranging from zero to 24. Scores of 0–8, 9–16, and 17–24 indicate “Poorly Satisfied”, “Satisfied”, and “Highly Satisfied”, respectively, with higher scores indicating greater patient satisfaction with nose surgery.^20^, ^21^, ^22^ Skin types were classified according to the Fitzpatrick scale (Types II, III, IV, V), describing pigmentation and response to ultraviolet exposure. Any complications that occurred during the study were recorded.

### Data analysis

Statistical analyses were performed using SPSS version 27. Categorical variables were reported as frequencies and percentages, while continuous variables were described with means and standard deviations if normally distributed, or medians with Interquartile Ranges (IQR) for non-normally distributed data. Changes in VAS and ROE scores during the postoperative periods were evaluated using appropriate statistical tests. The Friedman test was used for non-normally distributed data between paired groups, followed by Durbin–Conover pairwise comparisons. The Mann–Whitney test was applied for subgroup analysis by gender and operation type, while the Kruskal–Wallis test was used for comparisons by skin type. Statistical significance was set at p < 0.05.

## Results

### Characteristics of the included population

A total of 17 patients were enrolled in the study with a mean age of 28.9-years (SD = 7.68). The majority of patients were female (58.8%), with skin type III being the most common (47.1%) (Table 1).

**Table 1:** Patient demographics and clinical characteristics.

Age, mean (SD)	28.9 (SD = 7.68)	
Gender		
Female	10	58.80%
Male	7	41.20%
Operation type		
Primary	13	76.5%
Revision	4	23.5%
Skin Type		
II	1	5.9%
III	8	47.1%
IV	6	35.3%
V	2	11.8%

### Clinical evaluations (ROE and VAS)

Clinical assessments were performed at three postoperative visits: at a median of 4-weeks (IQR1), 9-weeks (IQR2), and 17-weeks (IQR3). Median ROE scores increased significantly from 16 (IQR4) on the first visit to 18 (IQR3) on the second visit, reaching 22 (IQR2) by the third visit. Moreover, the median VAS scores for the infratip, tip-defining point, and supratip areas showed marked reductions across the visits. In the infratip region, the scores decreased from 4 at the first visit to 3 at the second visit and further to 2 at the third visit (p < 0.001). Similar significant trends were observed in the tip-defining point and supratip areas (p < 0.05 for all pairwise comparisons). Detailed numerical summaries are provided in Table 2 and Figure S1.

**Table 2:** Summary of visit evaluations for ROE and VAS scores.

Visit (Weeks)	Timing (Weeks)	ROE	VAS (Infratip)	VAS (Tip Defining Point)	VAS (Supratip)
1st Visit	4 (IQR 1)	16 (4)	4 (3)	3 (2)	5 (3)
2nd Visit^a^	9 (IQR 2)	18 (3)	3 (2)	2 (1)	3 (2)
3rd Visit^b^	17 (IQR 3)	22 (2)	2 (1)	1 (1)	2 (1)
Overall p-value (Friedman test)		<0.001	<0.001	<0.001	<0.001
Pairwise Comparisons (Durbin-Conover)		P1 < 0.001; P2<0.001; P3 < 0.001	P1 < 0.001; P2<0.001; P3 < 0.001	P1 < 0.001; P2 <0.001; P3 < 0.001	P1 = 0.015; P2 < 0.001; P3 <0.001

Data are presented as median (IQR, Q3‒Q1).

Note: ^a^ 20 mg in 12 patients and 40 mg in 5 patients; ^b^2 patients needed no further punctures, 10 mg in 1 patient, 20 mg in 14 patients; Pairwise comparisons for ROE and VAS scores were conducted as follows: P1 represents comparisons between Visit 1 and Visit 2, P2 represents comparisons between Visit 1 and Visit 3, and P3 represents comparisons between Visit 2 and Visit 3.

ROE, Rhinoplasty Outcome Evaluation; VAS, Visual Analog Scale.

### Subgroup analyses

Subgroup analyses were performed to evaluate outcomes by gender, operation type, and skin type (see Appendix Tables S1, S2, and S3). The median ROE and VAS scores were comparable between male and female patients at all endpoints (all p > 0.05). Moreover, the only significant differences between primary and revision surgeries were observed at the first visit for both the ROE and infratip VAS scores (p = 0.03 and 0.02). No differences were noted at later visits. Furthermore, at all visits, there were no significant differences between median ROE or VAS scores across skin types (II, III, IV, and V) (all p-values > 0.05).

### Safety profile

The FAN technique for administering TA showed a strong safety profile, with no major adverse events reported. Participants experienced no pigmentation changes, significant skin reactions, or systemic complications like hypersensitivity or corticosteroid-related effects. While the injection process was described as briefly uncomfortable, the pain resolved spontaneously within minutes without requiring intervention.

## Discussion

Rhinoplasty is one of the most commonly performed aesthetic and functional facial procedures, yet postoperative challenges ‒ particularly in patients with thick nasal skin ‒ remain a significant concern. The presence of excessive fibro-fatty tissue in such patients can lead to prolonged edema, suboptimal definition, and an increased risk of scar tissue formation, ultimately affecting surgical outcomes and patient satisfaction.^23^ Corticosteroids, particularly TA, have been widely used in plastic and reconstructive surgery to mitigate these postoperative complications due to their potent anti-inflammatory properties, suppression of fibroblast activity, and inhibition of collagen synthesis.^24^^,^^25^ However, traditional administration methods often involve multiple punctures, which can contribute to discomfort, patient apprehension, and a higher risk of complications such as skin atrophy or telangiectasia. The FAN technique, employed in this study, represents an innovative approach aimed at maximizing the therapeutic benefits of TA while minimizing procedural drawbacks.

This study underscores the potential role of TA injection via the FAN technique in enhancing post-rhinoplasty recovery. The use of a single, strategically placed needle puncture at the nasal tip allowed for a controlled, widespread subcutaneous delivery of the medication. Initial intraoperative administration of 40 mg of TA effectively curtailed early postoperative swelling, with subsequent titrated doses (ranging from 10 mg to 40 mg) tailored to individual patient needs. By the third visit, two patients no longer required additional punctures, suggesting that an calibrated TA regimen may be sufficient to achieve sustained benefits in selected cases.

Patient-reported outcomes were significantly improved, as reflected in the ROE scores. The progressive increase in ROE across follow-up visits indicates a correlation between reduced inflammation, improved nasal contour, and enhanced patient satisfaction. These findings align with prior research by Baser et al., who reported a 76.92% satisfaction rate at one-year post-surgery in patients receiving targeted TA punctures.^26^ Additionally, median VAS scores for swelling in key anatomical regions ‒ specifically, the infratip, tip-defining point, and supratip × improved significantly (all p < 0.05), supporting the hypothesis that TA plays a crucial role in modulating postoperative edema.

While previous studies have suggested that the benefits of corticosteroid administration are transient,^27^^,^^28^ our findings indicate that a well-structured, patient-specific TA regimen may yield prolonged improvements. Saedi et al. noted only a temporary reduction in supratip edema at one-month post-surgery following TA injection^29^; however, improvements persisted beyond this period in our study, suggesting that the FAN technique may enhance drug distribution and retention within the tissue. Furthermore, subgroup analysis revealed that neither gender nor skin type significantly influenced postoperative ROE or VAS scores. Although initial differences in ROE and infratip VAS scores were observed between primary and revision rhinoplasty cases, these disparities diminished in later follow-ups. This suggests that TA injection via the FAN technique is broadly applicable across different patient demographics and surgical contexts.

One of the major advantages of the FAN technique is its ability to minimize patient discomfort while ensuring effective drug delivery. Traditional methods often require multiple injection points, increasing the risk of bruising, vascular injury, and patient distress. In contrast, the FAN technique’s single puncture approach appears to reduce these risks while maintaining efficacy. In our sample, no patients exhibited significant bruising or hematoma, supporting the presumed safety benefits of the single puncture technique employed in the FAN method; however, larger studies are required to confirm these findings conclusively. The most common adverse effect was transient, mild injection-site pain, which resolved without intervention.

Despite the promising findings, this study has several limitations. First, the small sample size limits the generalizability of the results, necessitating larger, multi-institutional studies for more robust conclusions. Second, the absence of a control group precludes direct comparisons with alternative treatment protocols, such as multiple-site TA punctures or other anti-inflammatory interventions. Consequently, it is difficult to definitively attribute the observed reduction in postoperative swelling solely to the TA infiltration, as natural postoperative healing processes may also have contributed to edema resolution. This limitation underscores the need for future double-blind, randomized controlled trials including a control arm without TA infiltration to establish the comparative efficacy of the FAN technique versus traditional methods. Additionally, our study relied primarily on patient-reported outcome measures, which, while valuable, introduce an element of subjectivity. The integration of objective assessments ‒ such as three-dimensional imaging to quantify edema and dermal thickness ‒ would strengthen the validity of future research. Furthermore, more extended follow-up periods are essential to assess the durability of TA’s effects, particularly in preventing hypertrophic scarring and late-stage fibrotic changes.

### Clinical implications

Our findings suggest that TA injection via the FAN technique is a viable adjunct in managing postoperative edema and optimizing outcomes in rhinoplasty patients with thick nasal skin. The combination of a minimally invasive administration approach with a tailored TA regimen offers a balance between efficacy and safety. By reducing the need for multiple punctures, this technique not only enhances patient comfort but also potentially lowers the risk of procedural complications.

## Conclusion

In conclusion, the FAN technique appears to be a promising advancement in post-rhinoplasty care. By facilitating improved swelling control, enhancing nasal contour, and boosting patient satisfaction across different demographics and surgical types, it may represent an important addition to contemporary rhinoplasty protocols. Future studies with larger sample sizes, objective imaging assessments, and extended follow-up durations will be instrumental in further validating its long-term efficacy and optimizing its application in clinical practice.

## ORCID ID

Ali Abdullah Alshehri: 0000-0002-9344-3825

## CRediT authorship contribution statement

AAA was solely responsible for the study design, methodology, data collection, data analysis, and interpretation of the results. AAA also wrote and approved the manuscript.

## Ethical considerations and consent to participate

Ethical approval for this study was granted by the Najran University Research Ethics Committee (Approval nº 202409-076-023707-053092). The research adhered to the ethical guidelines established by the Helsinki Declaration of 1964, as revised in October 2013. Informed consent was obtained from the parents or guardians of all participating pediatric patients prior to the study. Anonymity and confidentiality were maintained throughout the study, including data collection and analysis.

## Funding

None to declare.

## Consent for publication

Not applicable.

## Data availability statement

All data relevant to the study are included in the article or uploaded as supplemental information. The raw data supporting this study’s findings are available from the corresponding author upon reasonable request.

## Declaration of competing interest

The authors declare no conflicts of interest.
