# Supplementary material for: Triamcinolone acetonide for aesthetic refinement in rhinoplasty for patients with thick skin: the FAN technique ‒ a pilot study
Source: Braz J Otorhinolaryngol. 2026 Mar 9;92(3):101786. doi: 10.1016/j.bjorl.2026.101786 (PMC12994017; doi:10.1016/j.bjorl.2026.101786)
Supplement: Supplementary file 1 [file mmc1.docx]

**BJORL-D-25-00097**

**Supplementary Material**

**Appendix Table S1** ROE and VAS score analysis by gender.

|  | **N** | **Female (n = 10)** | **Male (n = 7)** | **Test Statistic** |
| --- | --- | --- | --- | --- |
| **Rhinoplasty Outcome Evaluation** | | | |  |
| 1^st^ Visit | 17 | 14.0 **17.0** 18.0 | 16.0 **16.0** 18.0 | F_1,15_ =0.20, p=0.66^a^ |
| 2^nd^ Visit | 17 | 17.8 **19.0** 20.2 | 16.0 **18.0** 20.0 | F_1,15_=0.35, p=0.57^a^ |
| 3^rd^ Visit | 17 | 19.8 **22.0** 22.0 | 20.3 **22.0** 22.0 | F_1,15_=0.46, p=0.51^a^ |
| **VAS (Infratip)** | |  |  |  |
| 1^st^ Visit | 17 | 3.0 **4.5** 6.1 | 3.0 **4.0** 5.8 | F_1,15_=0.29, p=0.60^a^ |
| 2^nd^ Visit | 17 | 2.0 **3.0** 4.1 | 2.0 **2.0** 3.8 | F_1,15_=0.74, p=0.40^a^ |
| 3^rd^ Visit | 17 | 1.0 **2.0** 2.0 | 1.0 **1.0** 2.0 | F_1,15_=1.04, p=0.32^a^ |
| **VAS (Tip Defining Point)** | | |  |  |
| 1^st^ Visit | 17 | 3.0 **3.5** 5.1 | 2.2 **3.0** 3.8 | F_1,15_=1.15, p=0.30^a^ |
| 2^nd^ Visit | 17 | 0.9 **2.0** 2.1 | 1.0 **1.0** 2.0 | F_1,15_=0.25, p=0.62^a^ |
| 3^rd^ Visit | 17 | 0.0 **1.0** 2.0 | 0.0 **0.0** 1.0 | F_1,15_=2.90, p=0.11^a^ |
| **VAS (Supratip)** | |  |  |  |
| 1^st^ Visit | 17 | 3.0 **5.0** 7.1 | 4.2 **5.0** 6.8 | F_1,15_=0.23, p=0.64^a^ |
| 2^nd^ Visit | 17 | 3.0 **4.0** 5.0 | 2.2 **3.0** 4.0 | F_1,15_=0.64, p=0.44^a^ |
| 3^rd^ Visit | 17 | 2.0 **2.5** 4.0 | 1.0 **2.0** 2.8 | F_1,15_=2.72, p=0.12^a^ |

N is the number of non-missing value.

^a^ Wilcoxon rank-sum test.

VAS, Visual Analog Scale.

**Appendix Table S2** ROE and VAS score analysis by operation type.

|  | **N** | **Primary (n=13)** | **Revision (n=4)** | **Test Statistic** |
| --- | --- | --- | --- | --- |
| **Rhinoplasty Outcome Evaluation** | | | |  |
| 1^st^ Visit | **17** | **14.0 16.0 18.0** | **18.0 18.0 19.2** | **F_1,15_=5.80, p=0.03^a^** |
| 2^nd^ Visit | 17 | 16.0 **18.0** 20.0 | 18.8 **20.0** 21.2 | F_1,15_=1.88, p=0.19^a^ |
| 3^rd^ Visit | 17 | 20.0 **22.0** 22.0 | 17.3 **22.0** 22.0 | F_1,15_=0.02, p=0.90^a^ |
| **VAS (Infratip)** | |  |  |  |
| 1^st^ Visit | 17 | **3.0 4.0 5.0** | **4.8 6.5 7.6** | **F_1,15_=6.64, p=0.02^a^** |
| 2^nd^ Visit | 17 | 2.0 **3.0** 4.0 | 2.4 **3.5** 6.9 | F_1,15_=0.78, p=0.39^a^ |
| 3^rd^ Visit | 17 | 1.0 **1.0** 2.0 | 1.4 **2.0** 5.5 | F_1,15_=1.99, p=0.18^a^ |
| **VAS (Tip Defining Point)** | | |  |  |
| 1^st^ Visit | **17** | **2.7 3.0 5.0** | **3.0 3.5 5.2** | **F_1,15_=0.41, p=0.53^a^** |
| 2^nd^ Visit | 17 | 1.0 **2.0** 2.0 | 0.8 **2.0** 4.9 | F_1,15_=0.27, p=0.61^a^ |
| 3^rd^ Visit | 17 | 0.0 **1.0** 1.0 | 0.0 **1.0** 3.8 | F_1,15_=0.22, p=0.65^a^ |
| **VAS (Supratip)** | |  |  |  |
| 1^st^ Visit | 17 | 4.0 **5.0** 7.3 | 3.4 **5.0** 6.6 | F_1,15_=0.20, p=0.66^a^ |
| 2^nd^ Visit | 17 | 3.0 **3.0** 5.0 | 2.4 **3.5** 4.6 | F_1,15_=0.12, p=0.74^a^ |
| 3^rd^ Visit | 17 | 1.0 **2.0** 3.0 | 2.0 **2.5** 4.2 | F_1,15_=0.99, p=0.33^a^ |

N is the number of non-missing value.

^a^ Wilcoxon rank-sum test.

VAS, Visual Analog Scale.

**Appendix Table S3** ROE and VAS score analysis by skin type.

|  | **N** | **II (n=1)** | **III (n=8)** | **IV (n=6)** | **V (n=2)** | **Test Statistic** |
| --- | --- | --- | --- | --- | --- | --- |
| **Rhinoplasty Outcome Evaluation** | | | | | | |
| 1^st^ Visit | 17 | 14.0 **14.0** 14.0 | 14.0 **17.0** 19.2 | 15.8 **17.0** 18.0 | 16.0 **17.0** 18.0 | F_3,13_=0.41, p=0.75^a^ |
| 2^nd^ Visit | 17 | 20.0 **20.0** 20.0 | 16.0 **19.0** 22.0 | 15.7 **19.0** 20.0 | 18.0 **18.0** 18.0 | F_3,13_=0.25, p=0.86^a^ |
| 3^rd^ Visit | 17 | 22.0 **22.0** 22.0 | 20.0 **22.0** 22.0 | 19.5 **21.0** 22.0 | 22.0 **22.0** 22.0 | F_3,13_=0.64, p=0.60^a^ |
| **VAS (Infratip)** | | | | | | |
| 1^st^ Visit | 17 | 5.0 **5.0** 5.0 | 3.0 **3.5** 5.6 | 3.9 **5.5** 6.2 | 3.0 **3.5** 4.0 | F_3,13_=0.89, p=0.471 |
| 2^nd^ Visit | 17 | 4.0 **4.0** 4.0 | 2.0 **3.0** 3.6 | 2.0 **3.5** 6.2 | 2.0 **2.0** 2.0 | F_3,13_=1.23, p=0.341 |
| 3^rd^ Visit | 17 | 2.0 **2.0** 2.0 | 1.0 **1.0** 2.0 | 1.0 **2.0** 2.5 | 1.0 **1.5** 2.0 | F_3,13_=0.82, p=0.511 |
| **VAS (Tip Defining Point)** | | | | | | |
| 1^st^ Visit | 17 | 3.0 **3.0** 3.0 | 2.4 **3.0** 5.0 | 3.0 **3.5** 6.0 | 2.0 **3.0** 4.0 | F_3,13_=0.44, p=0.731 |
| 2^nd^ Visit | 17 | 2.0 **2.0** 2.0 | 0.4 **1.5** 2.0 | 1.0 **2.0** 6.1 | 1.0 **1.5** 2.0 | F_3,13_=0.55, p=0.661 |
| 3^rd^ Visit | 17 | 1.0 **1.0** 1.0 | 0.0 **0.5** 1.6 | 0.0 **0.5** 1.3 | 1.0 **1.5** 2.0 | F_3,13_=0.48, p=0.701 |
| **VAS (Supratip)** | | | | | | |
| 1^st^ Visit | 17 | 5.0 **5.0** 5.0 | 3.4 **5.0** 7.6 | 4.0 **5.5** 7.1 | 3.0 **4.5** 6.0 | F_3,13_=0.18, p=0.911 |
| 2^nd^ Visit | 17 | 5.0 **5.0** 5.0 | 3.0 **3.0** 5.0 | 2.9 **4.0** 5.2 | 2.0 **2.5** 3.0 | F_3,13_=1.12, p=0.381 |
| 3^rd^ Visit | 17 | 3.0 **3.0** 3.0 | 1.4 **2.0** 3.2 | 1.0 **2.5** 3.2 | 2.0 **2.5** 3.0 | F_3,13_=0.23, p=0.881 |

N is the number of non-missing value.

^a^ Kruskal-Wallis test.

VAS, Visual Analog Scale.

**Figure S1** Summary of visit evaluations for ROE and VAS scores.
